# Multi-dimensional consistency learning between 2D Swin U-Net and 3D U-Net for intestine segmentation from CT volume

**DOI:** 10.1007/s11548-024-03252-6

**Published:** 2025-02-22

**Authors:** Qin An, Hirohisa Oda, Yuichiro Hayashi, Takayuki Kitasaka, Hiroo Uchida, Akinari Hinoki, Kojiro Suzuki, Aitaro Takimoto, Masahiro Oda, Kensaku Mori

**Affiliations:** 1 Graduate School of Informatics, Nagoya University, Nagoya, Aichi 4648601 Japan; 2 School of Management and Informatics, University of Shizuoka, Suruga-ku, Shizuoka 4228526 Japan; 3 School of Information Science, Aichi Institute of Technology, Toyota, Aichi 4700392 Japan; 4 Graduate School of Medicine, Nagoya University, Nagoya, Aichi 4668550 Japan; 5 Department of Radiology, Aichi Medical University, Nagakute, Aichi 4801195 Japan; 6 Information Technology Center, Nagoya University, Nagoya, Aichi 4648601 Japan; 7 Research Center for Medical Bigdata, National Institute of Informatics, 2-1-2 Hitotsubashi, Chiyoda-ku, Tokyo 1018430 Japan

**Keywords:** Intestine segmentation, Semi-supervision, Computer-aided diagnosis, Transformer

## Abstract

**Purpose:**

The paper introduces a novel two-step network based on semi-supervised learning for intestine segmentation from CT volumes. The intestine folds in the abdomen with complex spatial structures and contact with neighboring organs that bring difficulty for accurate segmentation and labeling at the pixel level. We propose a multi-dimensional consistency learning method to reduce the insufficient intestine segmentation results caused by complex structures and the limited labeled dataset.

**Methods:**

We designed a two-stage model to segment the intestine. In stage 1, a 2D Swin U-Net is trained using labeled data to generate pseudo-labels for unlabeled data. In stage 2, a 3D U-Net is trained using labeled and unlabeled data to create the final segmentation model. The model comprises two networks from different dimensions, capturing more comprehensive representations of the intestine and potentially enhancing the model’s performance in intestine segmentation.

**Results:**

We used 59 CT volumes to validate the effectiveness of our method. The experiment was repeated three times getting the average as the final result. Compared to the baseline method, our method improved 3.25% Dice score and 6.84% recall rate.

**Conclusion:**

The proposed method is based on semi-supervised learning and involves training both 2D Swin U-Net and 3D U-Net. The method mitigates the impact of limited labeled data and maintains consistncy of multi-dimensional outputs from the two networks to improve the segmentation accuracy. Compared to previous methods, our method demonstrates superior segmentation performance.

## Introduction

Intestine obstruction [1–3] is a serious disease often resulting from tumors and intestinal twisting. Computed tomography (CT) is a powerful technology offering detailed intestinal information, enabling clinicians to diagnose diseases by checking CT volumes. However, the process is time-consuming, given the hundreds of slices in a CT volume. Intestine segmentation helps diagnose intestinal diseases and aids in facilitating the development of treatment plans.

**Fig. 1:**
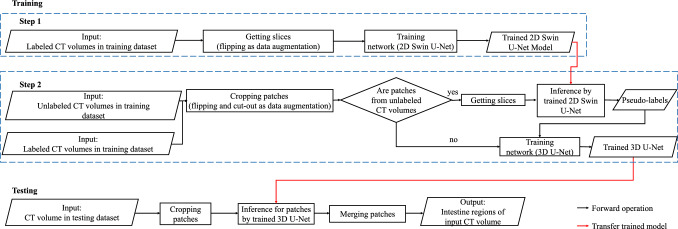
The flowchart of our method. For training, in step 1, we train a 2D Swin U-Net using labeled slices and then generate pseudo-labels using the trained model for unlabeled data. In step 2, cropped patches from both labeled and unlabeled data are used to train the 3D U-Net. For testing, we crop patches from the testing dataset and employ the trained 3D U-Net to infer these patches. Finally, we merge the inferred patches as the model’s output

Complex structure and contacting neighboring organs pose challenges for intestine segmentation. Currently, there are some thresholding-based methods [4–6] for organ segmentation, which mainly utilizes the intensity of the image. Full-supervision learning [7–10] is used for intestine segmentation. An obvious drawback of the full-supervision method is the substantial requirement for pixel-level labeled data to achieve satisfactory results. However, labeling medical images is time-consuming because it needs to be done by clinicians slice by slice.

For the limited labeled data problem, semi-supervision learning [11] has captured researchers’ attention in organ segmentation. Pseudo-labeling [12, 13] and consistency learning [14, 15] are primary strategies in semi-supervised learning. We introduce these strategies to intestine segmentation. The proposed method utilizes a 2D transformer generating pseudo-labels for unlabeled data, and then, a 3D convolutional neural network (CNN) is trained using the limited labeled data and ample unlabeled data with pseudo-labels. 2D Swin U-Net [16] is developed based on the vision transformer, which can capture long-range dependencies and enhance global contextual information by self-attention mechanism, improving the segmentation results of complex structures in medical images. 3D U-Net [17] is a classical network for medical segmentation that can effectively utilize the intra-slice and inter-slice features.

Qin, et al. [18] employed bidirectional teaching with two improved 3D U-Nets generating pseudo-labels for intestine segmentation. However, the pseudo-labels are unreliable since the networks with limited performances due to training with limited labeled data in the early stage of training. In contrast to this method [18], we train a 2D Swin U-Net with large-scale 2D slices from 3D CT volumes to generate pseudo-labels avoiding the pseudo-labels unreliable in the early stage of training and leverage the consistency learning between the transformer and CNN.

Our method trains a two-stage network and combines it with multi-dimensional consistency learning to segment intestines from CT volumes. The contributions of this paper are summarized as:

- We propose a novel two-stage network, which utilizes large-scale labeled slices to train a 2D Swin U-Net for generating pseudo-labels avoiding unreliable pseudo-labels generated by 3D networks with limited labeled data, and a 3D U-Net is trained using both labeled and unlabeled data, preventing the neglect of inter-slice features just using the 2D network.
- We use a multi-dimensional consistency learning for a new semi-supervision strategy, which not only effectively utilizes unlabeled data by pseudo-labels but also improves the model’s robustness by the consistency between segmentation results from 3D U-Net and pseudo-labels from 2D Swin U-Net by consistency learning.

## Method

### Overview

Our method aims to segment the intestine from CT volumes that train two networks in two steps. In step 1, we utilize labeled slices to train 2D Swin U-Net [16]. In step 2, we employ a limited number of labeled data and large-scale unlabeled data to train the 3D U-Net [17]. For the labeled data, we use a supervised loss function to update the model’s parameters. For the unlabeled data, firstly the trained 2D Swin U-Net is used to generate pseudo-labels. Then, we use an unsupervised loss function to calculate the loss keeping consistency between predictions of unlabeled data from 3D U-Net and corresponding pseudo-labels from 2D Swin U-Net. For testing, we use trained 3D U-Net to infer the patches cropped from the testing data and merge the patches to CT volumes as the final output. The flowchart of our method is shown in Fig. 1.

**Fig. 2:**
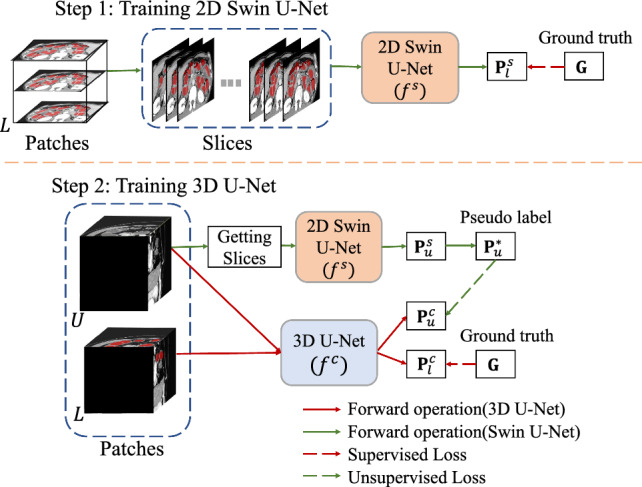
Structure of our method. Step 1 contains training of a 2D Swin U-Net and then using the trained 2D Swin U-Net to generate pseudo-labels for unlabeled data. Step 2 contains training of a 3D U-Net with the labeled and unlabeled data

**Fig. 3:**
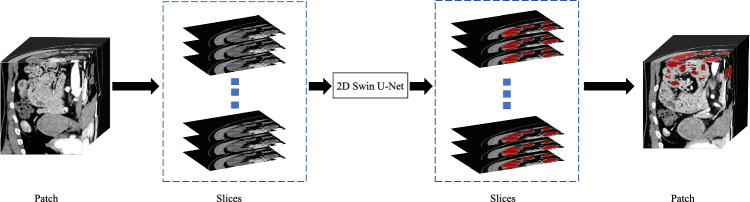
Getting slices operation from the patches and the process of generating pseudo-labels. We extract axial slices from the patch and infer them by 2D Swin U-Net. Then, pseudo-label for the patch is obtained by merging these slices. For example, one patch with size 256$\times$256$\times$16 can be divided into 16 slices with size 256$\times$256

**Fig. 4:**
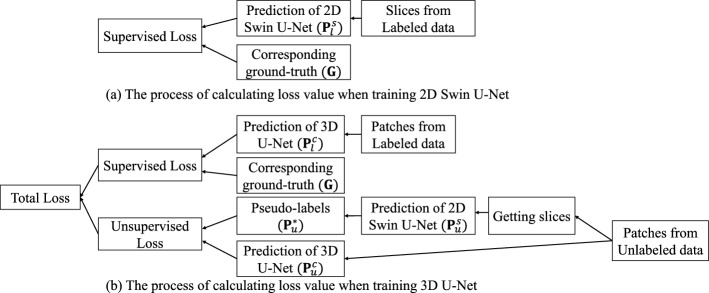
The process of calculating loss value. **a** and **b** show calculating loss when training 2D Swin U-Net and 3D U-Net

### Two-step network with multi-dimensional consistency learning

#### Two-step network

To improve the accuracy of intestine segmentation, we have develop a novel multi-dimensional consistency learning approach. In general, segmentation networks require an ample amount of labeled data to achieve good performance. Considering the use of limited labeled data to train a network that generates pseudo-labels, the resulting network may generate low-quality pseudo-labels due to poor performance. CT volume is a 3D image containing many 2D slices. Therefore, the proposed method utilizes 2D CT slices in the first step and 3D patches in the second step. The structure of the two-step network is shown in Fig. 2.

Two-step network contains two networks: 2D Swin U-Net $\left( f^{s}(\cdot ) \right)$ and 3D U-Net $\left( f^{c}(\cdot ) \right)$. 2D Swin U-Net is the first symmetrical U-shape network based on the transformer, implementing self-attention in the encoder. 3D U-Net is a classical medical image segmentation model for organs with relatively simple spatial structures. However, it exhibits inadequate intestine segmentation due to the intestine’s complex structure and limited labeled data.

The proposed network uses the slices from labeled data $({\textbf {D}}^l_{slice})$ and corresponding ground truth to train 2D Swin U-Net. Getting slices operation is shown in Fig. 3. Then, the trained model generates the pseudo-labels for the slices from unlabeled data $({\textbf {D}}^u_{slice})$

1 $$\begin{aligned} {\textbf {{P}}}^{s}_{u} = f^{s}({\textbf {D}}^u_{slice}), \end{aligned}$$

where ${\textbf {{P}}}^{s}_{u}$ represent 2D Swin U-Net’s $\left( f^{s}(\cdot ) \right)$ prediction result of unlabeled data. Note that the trained 2D Swin U-Net takes slices as input to get outputs, and we combine these outputs into a patch as the final output. Based on the prediction ${\textbf {{P}}}^{s}_{u}$, the pseudo-labels $({\textbf {P}}^{*}_{u})$ for the unlabeled data are generated by the argmax operation that converts probabilities to discrete class labels.

For the 3D U-Net, we directly use patches from labeled and unlabeled data as the input for training. The prediction of the 3D U-Net for the labeled and unlabeled data$~{\textbf {{P}}}^{c}_{l}$ and ${\textbf {{P}}}^{c}_{u}$ is represented by

2 $$\begin{aligned} {\textbf {{P}}}^{c}_{l} = f^{c}({\textbf {D}}^l_{patch}),~ {\textbf {{P}}}^{c}_{u} = f^{c}({\textbf {D}}^u_{patch}), \end{aligned}$$

where ${\textbf {D}}^l_{patch}$ and $~{\textbf {D}}^u_{patch}$ represent the 3D patches cropped from labeled and unlabeled data, respectively.

In multi-dimensional consistency learning, the two networks collaborate to enable the model to leverage the strengths of two different architectures, effectively improving the model’s learning ability and achieving better segmentation performance.

#### Multi-dimensional consistency learning

In the proposed method, the unsupervised loss is calculated using the predictions from 3D U-Net and the pseudo-labels from 2D Swin U-Net. Multi-dimensional consistency learning is used to maintain consistency between them. The process is represented by the green dashed lines in Fig. 2.

### Loss function

The proposed method involves training two networks, each corresponding to a different loss function. The 2D Swin U-Net is trained using a supervised loss function, while the 3D U-Net is trained using both supervised and unsupervised loss functions. The overview of calculated loss is shown in Fig. 4.

We just use supervised loss $L_{sup}$ to train a 2D Swin U-Net. The supervised loss consists of cross-entropy (CE) loss $L_{ce}$ and Dice loss $L_{dice}$

3 $$\begin{aligned} L_{sup} ({\textbf {{P}}}^{s}_{l},{\textbf {G}}) = \alpha L_{ce}({\textbf {{P}}}^{s}_{l},{\textbf {G}})+ (1-\alpha ) L_{dice}({\textbf {{P}}}^{s}_{l},{\textbf {G}}), \end{aligned}$$

where ${\textbf {{P}}}^{s}_{l}$ denotes the 2D Swin U-Net’s prediction result, and ${\textbf {{G}}}$ denotes the ground truth. We experimentally set the weight $\alpha$ to 0.3.

To train the 3D U-Net, we use the supervised loss $L_{sup}$ for labeled data and unsupervised loss $L_{un}$ for unlabeled data. The supervised loss is the same as for training 2D Swin U-Net. We just use Dice loss as unsupervised loss for the unlabeled data to avoid unstable training process due to the serious class imbalance.

4 $$\begin{aligned} L_{sup} ({\textbf {{P}}}^{c}_{l},{\textbf {G}}) = \alpha L_{ce}({\textbf {{P}}}^{c}_{l},{\textbf {G}})+ (1-\alpha ) L_{dice}({\textbf {{P}}}^{c}_{l},{\textbf {G}}), \end{aligned}$$

5 $$\begin{aligned} L_{un}({\textbf {{P}}}^{c}_{u},{\textbf {{P}}}^{*}_{u}) = L_{dice}({\textbf {{P}}}^{c}_{u},{\textbf {{P}}}^{*}_{u}), \end{aligned}$$

where$~{\textbf {{P}}}^{c}_{l}$ and ${\textbf {{P}}}^{c}_{u}$ represent 3D U-Net’s prediction results of labeled and unlabeled data, and ${\textbf {{P}}}^{*}_{u}$ represents pseudo-labels obtained from 2D Swin U-Net for ${\textbf {{P}}}^{c}_{u}$. The total loss for 3D U-Net is defined as

6 $$\begin{aligned} L_{total} \left( {\textbf {{P}}}^{c}_{l},{\textbf {G}}, {\textbf {{P}}}^{*}_{u},{\textbf {{P}}}^{c}_{u} \right) = L_{sup} \left( {\textbf {{P}}}^{c}_{l},{\textbf {G}} \right) + L_{un} \left( {\textbf {{P}}}^{*}_{u},{\textbf {{P}}}^{c}_{u} \right) . \end{aligned}$$

## Experiments and results

### Dataset and experimental setup

We used an intestine dataset consisting of 171 cases of ileus patients’ CT volumes with size 512 $\times$ 512 $\times$ (198–546) voxels, resolution (0.549–0.904 mm/voxels) $\times$ (0.549–0.904  mm/voxels) $\times$ (1.0–2.0 mm/voxels). These CT volumes were interpolated to isotropic voxel resolution ($\hbox {1mm}^3$/voxels). Interpolated volume sizes were (281$\times$281)–(463$\times$463) $\times$ (396–762) voxels. The training dataset with 85 CT volumes includes 13 densely labeled data and 72 unlabeled data. 27 sparsely labeled CT volumes were used for validation. Testing dataset with 59 CT volumes includes 58 sparsely labeled data and one densely labeled data for 3D visualization of a result. CT volumes that have labels of the intestine in some discontinuous slices are called sparsely labeled data. For one sparsely labeled data, with the percentage of labeled slices in one CT volume ranging from 1.00% to 5.31%, the number of labeled slices ranges from 6 to 29. CT volumes that have labels of the intestine in hundreds of continuous slices but not every slice were called densely labeled data. For one densely labeled data, the percentage of labeled slices in one CT volume ranges from 35.73% to 64.43%, and the number of labeled slices ranges from 154 to 319.

For training, we utilized a sliding window of size 256 $\times$ 256 $\times$ 16 with a stride of 128 $\times$ 128 $\times$ 8 to crop patches from the training dataset after the isotropic interpolation. We divided labeled patches (cropped from labeled data) into slices and applied flipping as data augmentation to generate training data for the 2D Swin U-Net. Labeled and unlabeled patches (cropping from unlabeled data) were used for training 3D U-Net, and flipping and cut-out were applied to them as data augmentation. We quantitatively evaluated the segmentation results using three metrics: 1) Dice; 2) recall; and 3) precision rates.

We conducted a series of experiments, including a contrasting experiment with previous methods (*Ex* 1), an ablation study of supervision loss (*Ex* 2), an experiment of changing the parameter in supervision loss (*Ex* 3), and an ablation study of selecting first and second models (*Ex* 4) to validate the performance of our method. All experiments were repeated three times with different random seeds for training, demonstrating the robustness of our model and proving that it performs well under different initializations. The averaged result of three times experiments was considered the final result for each testing case, and we calculated the average and standard deviation (SD) from the final results along all the testing cases (59 cases).

The *p* value by the Wilcoxon signed-rank test on the Dice score was calculated to prove the validation of our method. For the sparsely labeled data, these metrics were calculated only in labeled slices.

The proposed method was implemented using the PyTorch and executed on an NVIDIA A100 80 G GPU. We trained the model up to 500 epochs and used the early stopping when the best Dice score of validation remained unchanged for 30 epochs. The SGD optimizer was employed, and the poly learning rate strategy was used to adjust the learning rate with an initial value of 0.01.

### Results

**Fig. 5:**
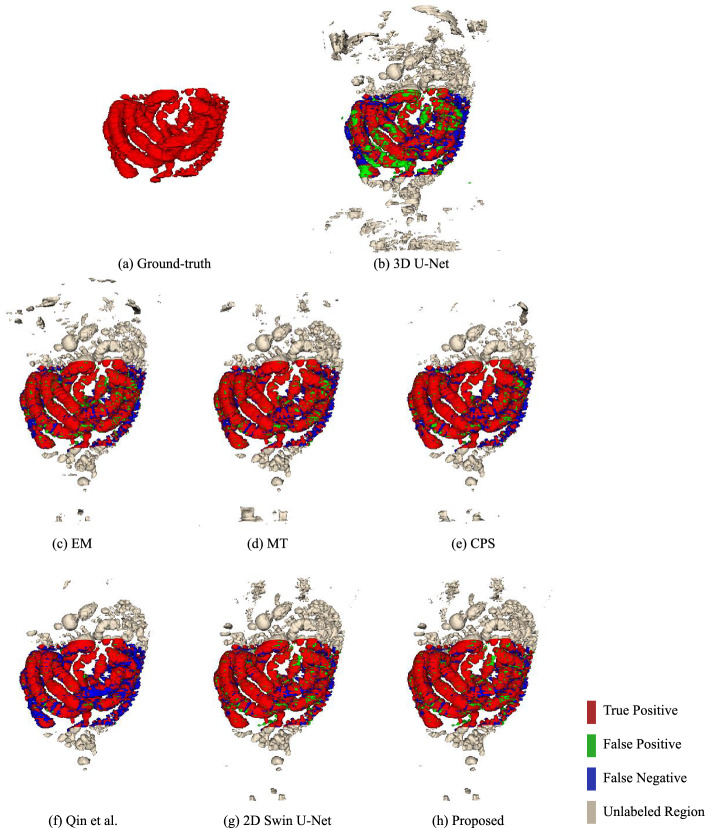
3D segmentation results from various methods. **a** is the ground truth; **b**–**h** are the results of different methods. The red, green, blue, and gray regions represent true positive, false positive, false negative, and the unlabeled regions, respectively

**Fig. 6:**
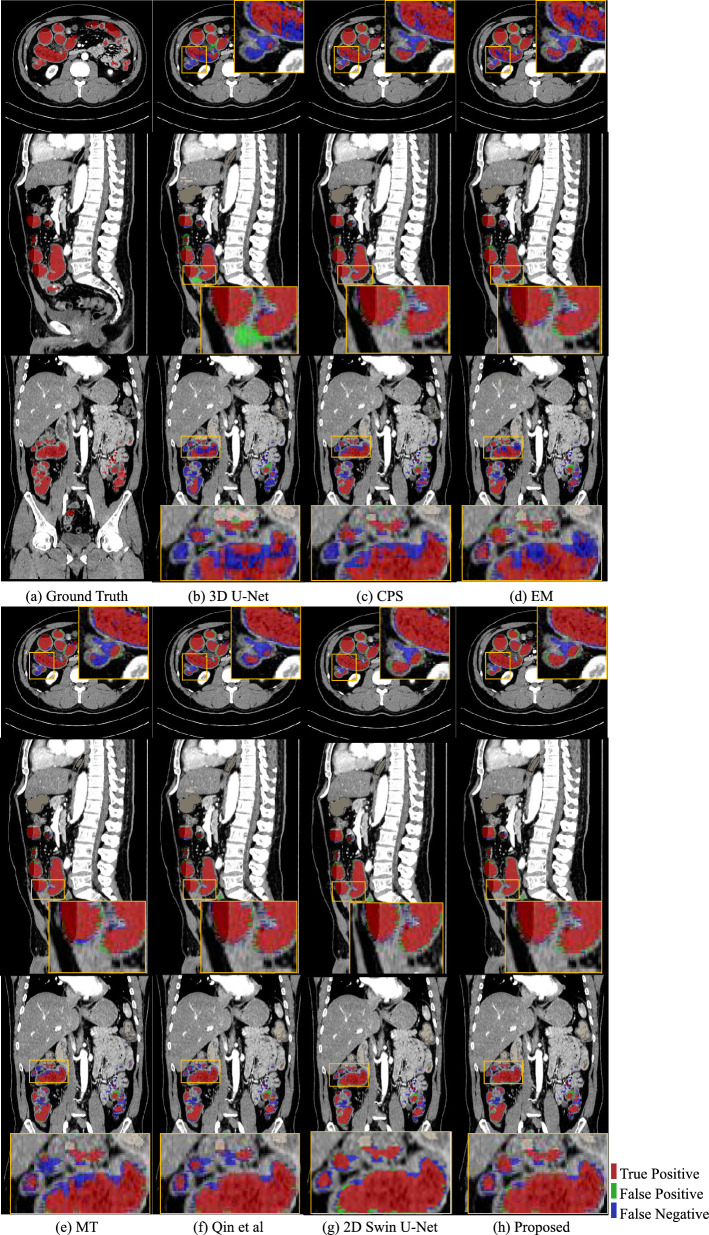
The 2D segmentation results of the different methods are displayed on three planes. The green color indicates false positives, and the blue color denotes false negatives. We can see that most mis-segmentation exists at the boundary part

**Fig. 7:**
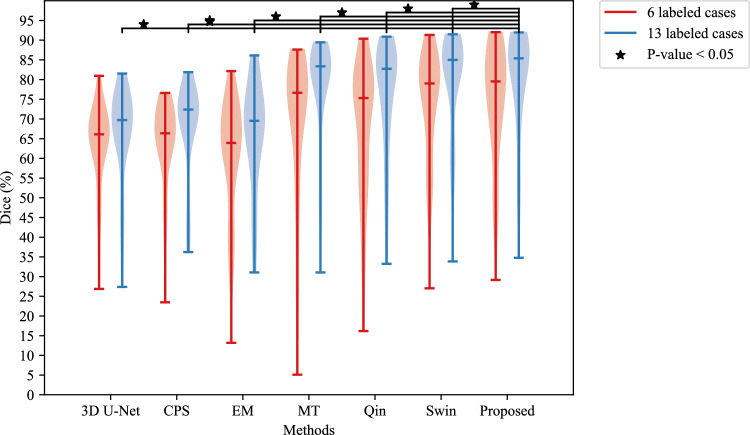
Violin plot of Dice score for different methods trained with 6 and 13 labeled cases. $\star$ denotes the *p* value based on the Wilcoxon signed-rank test < 0.05. Swin denotes 2D Swin U-Net

The quantitative results of *Ex* 1 are presented in Table 1, and we can see that the 81.75% of Dice score and the 7.65% of SD from the proposed method were the best performances. We conducted the Wilcoxon signed-rank test when the model was trained using 13 labeled cases, where the $\star$ denotes the *p* values were $<0.05$ among those methods. The segmentation results of *Ex* 1 are shown in Figs. 5 and 6. The results of training the proposed method using different number of labeled data are shown in Fig. 10. Figure 5 presents the 3D segmentation results, where red, green, and blue colors represent true positives, false positives, and false negatives, respectively. Since we utilize one densely labeled data to illustrate the 3D result, certain intestine regions lack labels in some slices. However, these methods can segment unlabeled intestine regions, depicted in gray. The 2D segmentation results are shown in Fig. 6. We can see from the zoomed regions in the yellow boxes that the proposed method improved the accuracy around the boundary. Figure 7 shows the distribution of Dice scores for each method on the testing dataset, and we calculated the *p* value when training with 13 labeled data, $\star$ means *p* values were $<0.05$ among those methods.

**Fig. 8:**
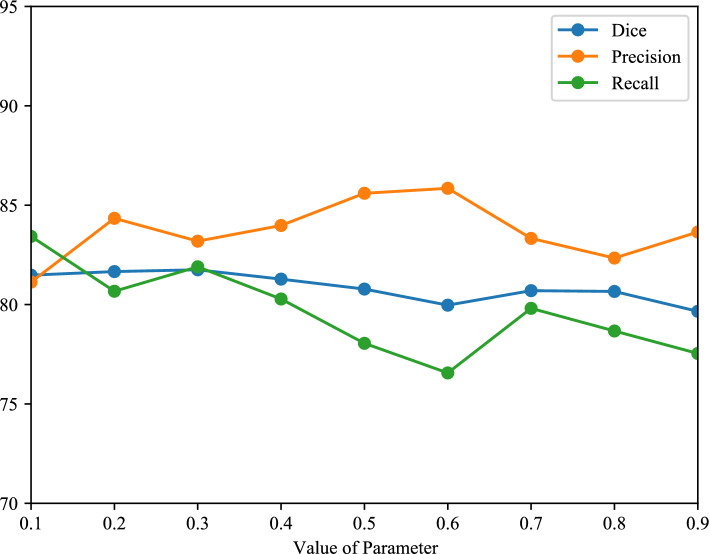
Line chart of qualitative results for different $\alpha$ in Eqs. (3) and (4). The horizontal axis represents the different parameters in the supervised loss. The vertical axis represents the results, and the blue, orange, and green lines denote Dice, precision, and recall rates, respectively

**Fig. 9:**
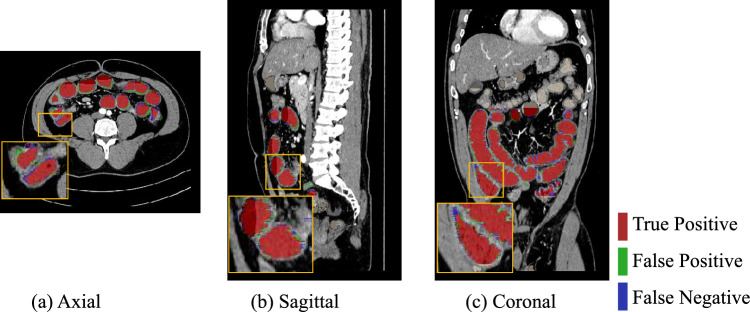
The 2D segmentation results of the proposed method are displayed on three planes. The red, green, and blue colors indicate true positives, false positives, and false negatives, respectively. Yellow boxes show zoomed images of the intestines

The results of *Ex* 2 are shown in Table 2, revealing that the proposed method with CE+Dice loss as the supervised loss function achieved the best result. The results of *Ex* 3 are shown in Fig. 8. We show the change in the Dice score, precision, and recall rates with blue, orange, and green colors, respectively. We can see that the best results are achieved when $\alpha =0.3$. Furthermore, the result of our method from three different planes is shown in Fig. 9. The results of *Ex* 4 are shown in Tables 3 and 4, revealing that the proposed method uses 2D Swin U-Net as the first step model and 3D U-Net as the second-step model achieved the best result in our intestine segmentation task.

**Fig. 10:**
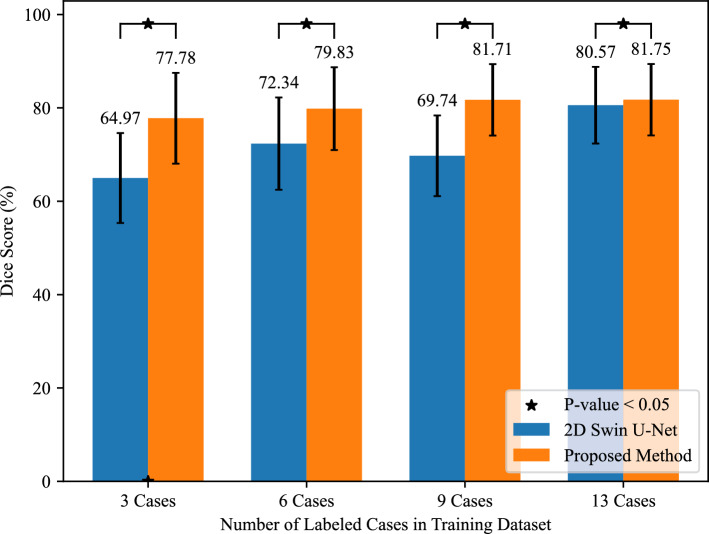
Bar chart of Dice score when there are different numbers of labeled cases in the training dataset to train 2D Swin U-Net and the proposed method. $\star$ denotes the *p* value based on the Wilcoxon signed-rank test < 0.05

**Table 1:** We compared the quantitative results of our proposed method with previous methods, including two full-supervised methods (3D U-Net, 2D Swin U-Net) and three semi-supervised methods (EM, MT, and CPS)

Labeled	Methods	Dice (%)	Precision (%)	Recall (%)
6 cases	3D U-Net [17]	44.63$\pm {13.08}$	55.93$\pm {21.53}$	44.02$\pm {13.01}$
	CPS [13]	68.94$\pm {12.05}$	**85**.**17**$\pm {10.74}$	61.90$\pm {13.28}$
	EM [19]	69.42$\pm {11.40}$	83.93$\pm {10.65}$	62.75$\pm {12.91}$
	MT [20]	71.60$\pm {10.84}$	83.30$\pm {11.36}$	65.97$\pm {12.16}$
	Qin et al. [18]	75.28$\pm {9.07}$	84.77$\pm {9.02}$	70.09$\pm {10.28}$
	2D Swin U-Net [16]	72.34$\pm {9.88}$	84.27$\pm {11.89}$	66.65$\pm {11.36}$
	Proposed	**79**.**83**$\pm {8.86}$	82.82$\pm {10.10}$	**79**.**04**$\pm {7.00}$
13 cases	3D U-Net	$48.48^\star \pm {10.86}$	80.42$\pm {11.44}$	40.68$\pm {9.88}$
	CPS	$77.54^\star \pm {8.86}$	85.31$\pm {8.78}$	73.60$\pm {9.73}$
	EM	$76.30^\star \pm {8.39}$	85.23$\pm {8.60}$	71.69$\pm {9.55}$
	MT	$76.82^\star \pm {8.03}$	85.76$\pm {8.79}$	71.93$\pm {8.89}$
	Qin et al.	$78.50^\star \pm {8.06}$	**85**.**88**$\pm {8.34}$	75.06$\pm {8.46}$
	2D Swin U-Net	$80.57^\star \pm {8.20}$	83.19$\pm {9.24}$	79.95$\pm {7.78}$
	Proposed	**81**.**75**$\pm {7.65}$	83.19$\pm {8.83}$	**81**.**90**$\pm {7.56}$

We calculate the *p* value on the Dice score between the proposed and previous methods, and $\star$ denotes the result $<0.05$. We highlight the best performance of each evaluation term with a bold font and show the SD

**Table 2:** To validate the effectiveness of the loss function, we use the different loss functions in the proposed method

Method	Dice (%)	Precision (%)	Recall (%)
Proposed with CE	80.32$\pm {8.07}$	**83**.**46**$\pm {9.11}$	79.15$\pm {7.75}$
Proposed with Dice	81.15$\pm {7.71}$	83.13$\pm {9.14}$	80.84$\pm {6.81}$
Proposed with CE+Dice	**81**.**75**$\pm {7.65}$	83.19$\pm {8.83}$	**81**.**90**$\pm {7.56}$

In these experiments, we use the same unsupervised loss function (Dice loss). We highlight the best performance of each evaluation term with a bold font

## Discussion

Our proposed method introduces multi-dimensional consistency learning for intestine segmentation. Firstly, in our method the 2D Swin U-Net was trained to generate pseudo-labels for unlabeled data, addressing the limited labeled data problem. Subsequently, we use limited labeled data and large-scale unlabeled data to train the 3D U-Net. For the unlabeled data, we use unsupervised loss to maintain consistency between pseudo-labels from the 2D Swin U-Net and the 3D U-Net’s prediction. A series of experiments have shown that our proposed method achieved competitive results.

The 3D and 2D segmentation results in Figs. 5 and 6 show that our method segmented more intestine regions. Since the proposed method employed unlabeled data by pseudo-labeling, consistency learning can effectively improve the segmentation results by reducing the effect of limited labeled data.

Table 1 indicates that the proposed method exhibits stable and competitive performance, characterized by a high Dice score and a low SD value. The 2D Swin U-Net showed higher quantitative results than the 3D U-Net, indicating that the 2D method outperformed the 3D network using limited labeled data. The 3D U-Net had the lowest Dice score because it was trained just using 13 labeled CT volumes, leading to underfitting, while the 2D Swin U-Net was trained using 3144 slices from 13 CT volumes. The 2D Swin U-Net was trained using sufficient data and generated more reliable pseudo-labels. Then, limited labeled data and large-scale unlabeled data, including reliable pseudo-labels, were used to train a 3D U-Net, which utilizes the advantages of two architectures, improving the network’s performance. Although our method slightly outperforms the 2D Swin U-Net with increased labeled data, The bar chart, in Fig. 10, shows the histogram of the Dice score when the 2D Swin U-Net and the proposed method were trained using different numbers of labeled cases in the training dataset. The result highlights our method’s suitability for tasks with few labeled cases. We also calculate the *p* value based on the Wilcoxon signed-rank test between the two methods and results < 0.05. Notably, our approach outperforms stand-alone 2D Swin U-Net and 3D U-Net models, underscoring the benefits of the extra dimension and pseudo-labels in enhancing model performance. Additionally, we compared our method with three classical semi-supervised methods (EM [19], MT [20], and CPS [13]), all using the 3D U-Net as their backbone.

EM makes the model more confident by reducing uncertainty in predicted class probabilities, encouraging definitive outputs. MT guides a student model with a teacher to ensure consistent learning from labeled and unlabeled data. CPS trains two models together, generating pseudo-labels for the other, leveraging consistency in predictions on unlabeled data. Our proposed method achieved the best results compared with them. In Fig. 7, $\star$ means the *p* value $<0.05$ when they were trained using 13 labeled data, which indicated the validity of the proposed method.

In Table 2, the ablation study about the loss function shows that the combination of the CE and Dice losses as the supervised loss achieved the best result, which compromises benefits from each loss function. In Fig. 8, we explored the effect of the parameter in the supervised loss when the parameter $\alpha = 0.3$ with a better result. The CE loss assigns higher likelihoods to the correct class, and the Dice loss evaluates both false positives and false negatives in the segmentation results. Combination of them as a loss function and experimentally setting appropriate ratios of them was conducive to improving segmentation accuracy.

We propose a two-step semi-supervised method based on the transformer and CNN two framework. In our method, the first step’s model is trained by labeled slices and generating pseudo-labels. Therefore, accuracy should be the primary concern. We chose three 2D transformer-based models (2D Swin U-Net, Trans U-Net, and UTNet) as candidates and trained them using 3144 labeled slices. The results in Table 3 show that the 2D Swin U-Net achieved the best Dice score and has a relatively small model size. Although the UTNet is the lightest model, it has the worst accuracy. TransUNet is the largest model but not the most accurate. Therefore, 2D Swin U-Net is the best model for the first step.

**Table 3:** Ablation studies different models as the first step

Model	Dice (%)	Precision (%)	Recall (%)	Weight Size (Mb)
UTNet	76.47$\pm {10.61}$	83.02$\pm {12.00}$	72.71$\pm {9.82}$	**56**
TransUNet	79.65$\pm {8.44}$	**83**.**51**$\pm {9.82}$	77.99$\pm {8.41}$	401
2D Swin U-Net	**80**.**57**$\pm {8.20}$	83.19$\pm {9.24}$	**79**.**95**$\pm {7.78}$	106

We use the same data to train another 2D transformer-based model as the first step. We highlight the best performance of each evaluation term with a bold font

**Table 4:** Ablation studies different models as the second step

Model	Dice (%)	Precision (%)	Recall (%)	Weight size (Mb)
U-Net	72.20$\pm {9.78}$	73.81$\pm {12.09}$	74.84$\pm {10.70}$	**7**
2D Swin U-Net	79.54$\pm {9.78}$	77.86$\pm {9.92}$	**83**.**63**$\pm {7.16}$	106
3D Swin U-Net	78.29$\pm {7.87}$	80.55$\pm {9.45}$	78.23$\pm {7.62}$	177
Swin UNetr	79.72$\pm {8.12}$	76.01$\pm {8.83}$	85.93$\pm {8.44}$	68
UNetr	74.36$\pm {7.96}$	83.07$\pm {10.75}$	69.63$\pm {8.47}$	356
Proposed (3D U-Net)	**81**.**75**$\pm {7.65}$	**83**.**19**$\pm {8.83}$	81.90$\pm {7.56}$	54

We use the 2D Swin U-Net as the first step and six different models as the second step to select the best one. We highlight the best performance of each evaluation term with a bold font

For the second step, we selected three 3D transformer-based models (3D Swin U-Net, Swin UNetr, and UNetr) and two 2D models (2D Swin U-Net, U-Net) and the 3D U-Net. We compared the accuracy and size of the models to select the best one. Table 4 shows that the best performance is achieved using the 3D U-Net as the second-step model. We argue that the other three 3D models have complex structures, requiring more labeled data to perform well in full-supervised learning tasks. In our approach, the second-step networks are trained with a small amount of labeled data and unlabeled data with pseudo-labels, a situation that does not take good advantage of these networks. Therefore, the 3D U-Net with a simple structure is more suitable as the second-step model. For the 2D models as the second step, when we use the 2D Swin U-Net as the second step, the model’s Dice score even slightly decreases compared to just using the 2D Swin U-Net. Although the 2D U-Net model is lightweight, it achieved low accuracy. Therefore, using 2D models as the second step is insufficient compared with the proposed methods for the intestine segmentation task.

In Fig. 9, we can see that some mis-segmentation still exists at the boundary part, which may be caused by intestines contacting neighboring organs in the boundary. The fine-tuning strategy may solve the problem.

## Conclusion

We propose a multi-dimensional consistency learning between 2D Swin U-Net and 3D U-Net to segment the intestine from CT volumes. The limited number of labeled data, complex structure, and contact with neighboring organs are great challenges for intestine segmentation. We design a two-stage network, and firstly, we train a 2D Swin U-Net to generate pseudo-labels for unlabeled data reducing the effect of the limited labeled data. Secondly, labeled and unlabeled data are used to train a 3D U-Net. The experimental results demonstrated good performances.

In the contrasting experiments, our method achieved the best performance in the intestine segmentation. Although the proposed method has achieved some results, there is still some mis-segmentation at the boundary part. In the future, we will focus on reducing the mis-segmentation in the boundary by using a fine-tuning strategy.
